# Social Group Differences in the Exposure Risk of COVID-19: A Case Study in Beijing, China

**DOI:** 10.3390/ijerph19031121

**Published:** 2022-01-20

**Authors:** Chen Lu, Xiaodi Yi, Xiaocui Ren

**Affiliations:** 1 School of Public Policy and Management, University of Chinese Academy of Sciences, Beijing 100190, China; lvchen@ucas.ac.cn; 2 Sino-Danish College, University of Chinese Academy of Sciences, Beijing 100190, China; yixiaodi19@mails.ucas.ac.cn

**Keywords:** COVID-19, exposure risk, group difference, health inequity

## Abstract

Taking Beijing as a case, this paper conducted a survey to collect the characteristics of residents’ daily activities, including the mode of frequency and duration of travel, the type and environment of activities, and the duration and frequency of activities. We calculated the COVID-19 exposure risk of residents in different activities based on the exposure risk formula; the influencing factors of residents’ exposure risk were analyzed by regression analysis. The variance of residents’ COVID-19 exposure risk was calculated by coefficient of variation. The main conclusions are as follows: (1) There are differences in activity types of COVID-19 exposure risk, which are survival activity, daily activity and leisure activity from high to low. (2) There are differences in populations of COVID-19 exposure risk. Education level, occupation and income are the main factors affecting residents’ COVID-19 exposure risk. (3) There is internal inequity in the risk of COVID-19 exposure. The exposure risk was higher on work days than on rest days. Health inequities at work are highest on both work days and rest days. Among the different population characteristics, male, 31–40 years old, married, with a high school education, income level of 20,001–25,000 yuan, with a non-local rural hukou, rental housing, farmers, three generations or more living together have a greater degree of COVID-19 exposure risk.

## 1. Introduction

### 1.1. Research Background

Since December 2019, the COVID-19 epidemic has become a world public health event, threatening the safety of human lives and bringing an unprecedented impact on the economic and social development of various countries [1]. In China, although COVID-19 has been under control overall, sporadic cases are still occurring, and the risk of localized outbreaks is of great concerns [2]. As the capital of China, Beijing is the center of national transportation. With more than 20 million permanent residents and a large scale of floating population, Beijing is under great pressure in the epidemic prevention. High density of population allocation makes the control situation more serious and complicated.

Studies show that the main route of transmission of novel coronavirus is respiratory droplet transmission and close contact transmission, and such transmission characteristics and asymptomatic infection have exposed everyone to the risk of COVID-19 infection [3]. However, because the disparity between different social group, individuals differ in their demographic characteristics and daily activity behaviors, which may also cause stratification phenomenon of the exposure risk for COVID-19. In other words, in the context of COVID-19, there may be social problems of health inequity among individuals.

### 1.2. Literature Review

Exposure refers to the factors that affect and may affect the occurrence of diseases and people’s health status, including individual characteristics, living habits, and various micro and macro environments on which human beings depend for survival [4]. Research on COVID-19 exposure has focused on healthcare workers, a high-risk exposure group [5,6,7,8]. Factors affecting COVID-19 exposure in healthcare workers include the nature of work, workplace, distance of contact with patients, and duration of exposure [5]. Among hospital workers, staff in isolation wards and fever clinics are at high risk for COVID-19 exposure, while administrative logistics staff and other positions in hospitals are generally at low risk for COVID-19 exposure. Overall, people engaged in COVID-19 related work are at higher risk [8,9,10]. In addition, studies have confirmed that having a health care worker in the family is associated with a higher risk of exposure [6].

As the focus of urban geography study, the research of residents’ spatiotemporal behavior fully considers the impact of daily human activities on urban planning and management [11]. In terms of the research content, spatiotemporal behavior research mainly focusses on activities such as residents’ commuting [12], shopping [13,14], leisure [15], and migration [16].

Margaret Whitehead believes that health equity refers to creating equal health opportunities to reduce health differences to the lowest possible level, namely, reflecting the goal of social justice in terms of population health [17]. The factors affecting health equity are very wide, mainly including income [18,19,20,21], occupation [22,23], education level [21,22], living conditions [23]. Furthermore, sociodemographic characteristics such as gender and age were also considered [18,24]. For measuring health inequality, there are mainly range method [25,26], Lorenz curve and Gini coefficient method [25], difference coefficient method [25]. During COVID-19, research on health inequities indicates that ethnic minorities are suffering higher rates of infection and death [26,27,28,29,30,31,32]. In addition, studies have shown that some vulnerable groups have suffered more social impact from COVID-19, such as unemployment [33,34].

Focusing research on COVID-19, while some have studied the risk of exposure to COVID-19 among healthcare workers, few have studied the risk of exposure to COVID-19 among the general public. Studies of the public have focused more on their rates of infection, death, or impact from COVID-19. Compared to the studies of globally influenced COVID-19, the studies of other epidemics such as SARS are less. When tracing the literature of other types of infectious diseases and exposure risks, we found rare studies about the exposure risk of the public. In fact, paying more attention to the exposure risk of public before infection can prevent more infections beforehand. Therefore, it is necessary to acknowledge which group of people has the most possibility to become infected before we take the anti-epidemic measurement and arrange limited resources. Previous studies focus either on particular group of people in the background of infectious disease, or on the inequity of social and economic status and public resource arrangement. The study of inequity of exposure risks to infectious diseases among different groups is rare. The varied exposure risk caused by different kinds of daily activities also deserve exploration.

Taking Beijing as an example, this paper aims to explore the risk of exposure to COVID-19 among different groups of peoples under the condition of uncertainty, and further study the inequities among different social groups concerning the health security. Firstly, we investigated the features of different daily activities among different social groups, which is also separately analyzed on work days and rest days. Secondly, we calculated the exposure risk of different activities by combining the residents’ spatiotemporal behavior and the surrounding environmental features. Thirdly, we calculated and compared the equity of the exposure risk to COVID-19 between different social groups. Our research may reveal the inequity of different social groups when it comes to the exposure risk to COVID-19 under uncertainty, and offer policymakers a reference when they are allocating anti-epidemic resources.

### 1.3. Study Area

This paper takes Beijing as a research city and uses questionnaire method to collect data of spatio-temporal behavior and social demographic information of Beijing residents.

Beijing is the world’s most populous national capital city, with over 21 million residents within an administrative area of 16,410.5 km^2^ [35]. It is located in Northern China and is governed as a municipality under the direct administration of the State Council with 16 urban, suburban, and rural districts. In 2020, Beijing’s annual GDP reached 3610.26 billion yuan [36].

Study have shown that inhalation is often the most relevant transmission mode in indoor environments [37]. Therefore, indoor COVID-19 prevention and control is particularly important. In February 2020, five government departments in Beijing jointly issued an epidemic prevention policy in public places, requiring people entering public places to wear masks and scan a health code to register [38]. This rule is strictly enforced in any indoor public place in Beijing. People will not be allowed to enter any indoor spaces without masks and a health code (a QR code that indicates whether people have visited high-risk areas).

## 2. Method

The method used in this paper is questionnaire survey, using firsthand data to reveal the inequity in COVID-19. The calculation methods of some core indicators are as follows.

### 2.1. Measure of Risk of Exposure to COVID-19


(1) $$\begin{array}{r} C_{Ex}=\underset{i\in \Phi^{w}}{\sum }\left(f_{i}^{x}\left(u,\theta ,\mu_{i}^{x},t_{i}^{x},\omega_{i}^{x}\right)+f_{i}^{y}\left(u,\theta ,v,t_{i}^{y},\mu_{i}^{y},\omega_{i}^{y}\right)\right)+\\ \underset{j\in \Phi^{r}}{\sum }\left(f_{j}^{x}\left(u,\theta ,\mu_{j}^{x},t_{j}^{x},\omega_{j}^{x}\right)+f_{j}^{y}\left(u,\theta ,v,t_{j}^{y},\mu_{j}^{y},\omega_{j}^{y}\right)\right) \end{array}$$

(2) $$f_{i}^{x}\left(u,\theta ,\mu_{i}^{x},t_{i}^{x},\omega_{i}^{x}\right)=c_{i,x}^{u}\left(u\right)c_{i,x}^{\theta }\left(\theta \right)\mu_{i}^{x}t_{i}^{x}\omega_{i}^{x}$$

(3) $$f_{i}^{y}\left(u,\theta ,v,t_{i}^{y},\mu_{i}^{y},\omega_{i}^{y}\right)=c_{i,y}^{u}\left(u\right)c_{i,y}^{\theta }\left(\theta \right)c_{i,y}^{\nu }\left(\nu \right)\mu_{i}^{y}t_{i}^{y}\omega_{i}^{y}$$

In the above formula, the first half of the summation formula calculates the risk of exposure on work days, and the second half represents the risk of exposure on rest days, both of which consist of activity exposure and travel exposure. Among them, u: activity venue/coefficient of the environment during travel process, namely indoor/outdoor; θ: activity venue/coefficient of the number of surrounding people in the travel environment; μ: coefficient of the mobility of people around the activity venue; $t_{i}^{x}$ represents the length of time residents stay at the activity venue; $\omega_{i}^{x}$ represents the frequency with which residents engage in a particular activity during work days or rest days; v: The mode residents travel to engage in a certain activity; $t_{i}^{y}$ represents the amount of time that residents spend on a certain mode of travel; $\mu_{i}^{y}$ represents the coefficient of mobility of people in a certain mode of travel; $\omega_{i}^{y}$ represents the travel frequency, which is the same as the activity frequency in the research.

In the activity section, residents’ exposure risk is measured according to the nature of the environment in the activity, population density, mobility, time of duration, and frequency of activity. According to a study in Korea which compares the COVID-19 risk differences between the indoor activities and the outdoor activities, the risk of becoming COVID-19 infected of indoor activities is 19 times of that of outdoor activities without wearing any masks [39]. However, the real situation is more complex in Beijing because of the coercive mask-wearing policies. Study have shown that wearing a mask can reduce the risk of COVID-19 infection by 53% [40]. It is testified that if all the people wear masks, the risk of COVID-19 transmission is only 1.5%, and it rise to 5% when the virus carrier is wearing a mask and other people do not. The risk is 30% if the virus carries does not wear a mask and other people keep wearing the masks [41]. Due to Beijing’s strict indoor mask-wearing policy, we assume that nearly all people keep wearing masks according to the government’s requirement and only one third of them keep wearing mask outside. Then, in the situation of uncertainty, the weights of the risk of indoor activities and outdoor activities are 1 and 2, respectively. During activity, if mobility of people exists, there is a higher probability of infection, and the value is 2. If there is no mobility of the surrounding people, the risk is low, and the assigned value is 1. Population density and time of duration are assigned by the median value of the option data. In a constantly mobile environment, commuting activity belongs to outdoor environment by default and it has 100% mobility. Based on the actual situation in Beijing, the activity of picking up children belongs to outdoor environment by default, while the dining environment belongs to indoor environment.

In the travel section, residents’ exposure risk is determined by the modes of travel, travel time, nature of the environment, population density, mobility, and frequency. All travel processes are outdoor activities with mobility and are assigned a value of 2. Given the characteristics of COVID-19, the greater the population density, the greater the risk of infection. The number of people gathered around residents varies among different travel modes. About the population density (within a radius of 1.5 m), we conducted field investigation and averaged the data. Under normal circumstances, the population density on the subway is about 4 people; the population density on buses and company shuttle buses is about 3 people. However, commuting activity generally occurs during the morning and evening rush hours. The population density is greater during the ride process compared to other times. At this time, the population density on the subway is 8 people; the population density on buses and company shuttle buses is 6 people. In most cases, there are 2 people in private cars and taxis. The population density is usually 1 person for those who travel by bicycle, on foot, or by electric bicycle. The median value of the option data is directly taken as the calculation criteria for other factors such as travel time and frequency.

### 2.2. Measure of Inequity

Firstly, a concise statistical table is used to group the amount of COVID-19 exposure by demographic characteristics to present the distribution of the exposure risk of COVID-19 among different populations in the form of mean values, which reveals the characteristics of population differences. Secondly, the coefficient of variation, namely standard deviation/mean value, is used to reveal the specific status of inequity of COVID-19 exposure. In this section, we will calculate the coefficients of variation for the overall population, different demographic characteristics (gender, age), work days/rest days, and different activity types, respectively.

The formula of coefficient of variation is:

Coefficient of Variation = (standard deviation/mean value) × 100%(4)

### 2.3. Data Collection

According to the purpose of the study, we designed a structured questionnaire to collect data about daily activities of Beijing residents including those who live, work or study in Beijing.

The questionnaire mainly includes four parts: (1) demographic characteristics of residents, including gender, age, education level, marital status, occupation, income, hukou (hukou is a system of household registration used in China. A household registration record officially identifies a person as a permanent resident of an area and includes identifying information such as name, birthplace, gender. There are two types of Hukou: agricultural hukou and non-agricultural hukou), ownership of the house, family structure. (2) Residents’ activity types. In the survey, we divided the time of a week into Monday to Friday (work days) and Saturday to Sunday (rest days), and collected information related to residents’ activities from the above two dimensions. We analyzed the two parts separately. Residents’ daily activities are mainly divided into 8 types, including commuting, dining out, work, personal affairs, shopping, picking up kids, leisure, socializing. In addition, on rest days, home activities were added to the questionnaire to reflect the real-life status of residents. Although home activities can reflect the activity status of residents, they are not outdoor activities and will not be considered in the later calculation. (3) Systematic characteristics of activities. Systematic characteristics of activities include activity time, location, activity environment (indoor or outdoor, population density, personnel mobility), activity duration and activity frequency. (4) Systematic characteristics of travel. In this study, travel originates from activity needs, so travel system belongs to activity system. The systematic characteristics of travel include travel time, travel mode and travel duration. The frequency of travel is consistent with the frequency of activity. It should be noted that travel environment (indoor or outdoor, population density, mobility) and other data were not asked in the questionnaire but were obtained through practical tests and common sense.

We conducted questionnaires in November 2020 by both online interview and onsite interview. There are 756 interviewees, and finally, 754 valid questionnaires were obtained.

In terms of the number of questionnaires issued, we took the proportion of residents’ occupation distribution as a reference standard. From the Beijing Statistical Yearbook, the National Bureau of Statistics and relevant literature, we found information on the number of civil servants, employees in public institutions, employees in state-owned enterprises, employees in private enterprises, employees in foreign enterprises, individual business, freelancer, students, farmers, unemployed in Beijing in 2018 [42,43]. Based on the above data, we can obtain the proportion distribution of Beijing residents in these occupations. as According to the reference standard, we found the target population in proportion for targeted distribution and collection of questionnaires.

### 2.4. Sample Characteristics

**Figure 1:**
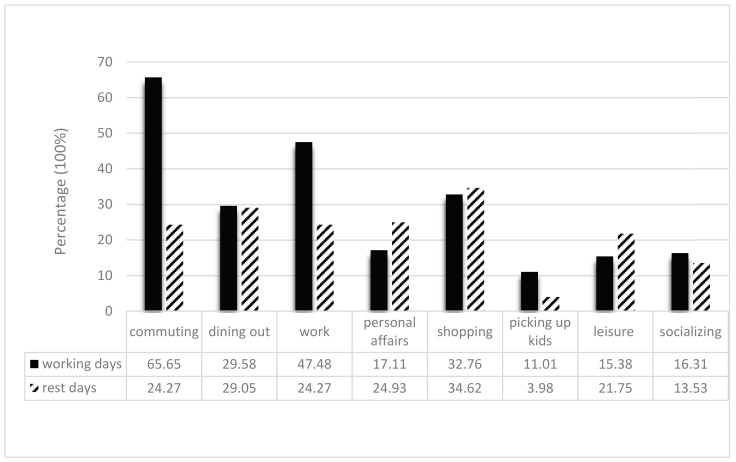
The percentage of different types of activities.

Descriptive statistics of the sample are shown in Table 1. The activity types of residents are shown in Figure 1. Commuting and work are the primary activities of residents on work days. Life activities such as shopping, dining out and personal affairs are the main contents of the rest days.

## 3. Results

### 3.1. Differences in COVID-19 Exposure Risk Stem from Different Activities

The difference of activity exposure risk on weekdays and rest days with and without activity frequency is shown in Table 2. By adding the exposure risk values for work days and rest days (considering the frequency of activity), we can obtain the total exposure risk for each activity over a week (see Table 3). Working and commuting activities composite the highest COVID-19 exposure risk during work days. Working and leisure activities contribute to the most exposure risk in rest days. In a whole week, working, commuting, and dining out activities are the top three activities that lead to exposure risks.

Different types of activities could be summarized into three categories, according to the nature of each activity. Based on the total exposure value of each activity in one week, the exposure risk of residents’ activities has the following characteristics: Survival activities had the highest exposure risk, followed by life activities and leisure activities. And the exposure risks for the three activity types are shown in Table 4.

### 3.2. Differences in COVID-19 Exposure Risk of Different Social Groups

The exposure risk levels of different social groups are shown in Table 5. It shows that residents in Beijing present varying characteristics of COVID-19 exposure risks among different social groups.

Based on the statistical results of different social groups, we further applied one-way ANOVA after winsorization to analyze whether the above sociodemographic characteristics have statistically significant differences in their impact on exposure risk. The results in Table 6 showed that, among the above demographic attributes, only education level, occupation and income had statistical significance on exposure risk level.

We took the total exposure risk of residents as the dependent variable, and the education level, occupation and income that passed the single factor analysis as the independent variables and applied multiple linear regression to analyze the relationship between the above three factors and the exposure risk of residents. Through a statistical test, the variance inflation factor (VIF) of these independent variables is less than 10, so there is no serious collinearity. The regression results are shown in Table 7.

Those with education levels of high school (*p* < 0.01), junior college (*p* < 0.1) and undergraduate (*p* < 0.05) were at higher risk of COVID-19 exposure compared to those with education levels of junior high school and below. Compared to civil servants, the student group was at greater risk of exposure (*p* < 0.05). Other occupational groups failed the significance test. Those with a monthly income level of ≥30,001 yuan were at greater risk of exposure compared to those with a monthly income of ≤2000 yuan (*p* < 0.01).

### 3.3. Inequity of Exposure Risk

We further analyzed inequity of COVID-19 exposure risk using coefficient of variation. Coefficient of variation less than 0.1 represents weak variation, between 0.1 to 1 is moderate variation, and ≥1 means strong variation [44]. The total coefficient of variation is 1.46, which means there are huge differences of COVID-19 exposure risk among different social groups. The detailed inequity results in each group are shown in Table 8. 

**Figure 2:**
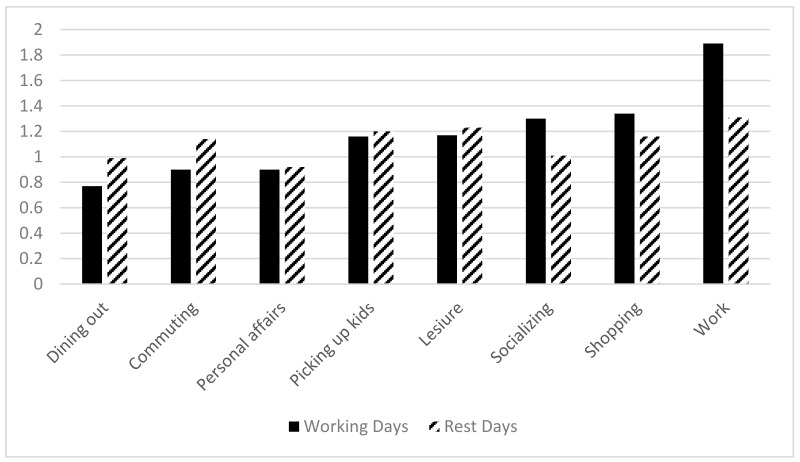
Coefficient of variation of different activities.

The degree of health inequality on work days (1.64) is higher than that on rest days (1.31). There are health inequalities between different activities. From the perspective of activity type, on work days, the degree of health inequity at work is the highest (1.94), while the degree of inequity at dining out is the lowest (0.79). On rest days, the degree of health inequity at work is the highest (1.34), while the degree of inequity at personal affairs is the lowest (0.92). The coefficient of variation for all activities are shown in Figure 2.

Health inequality also varies among people with different socioeconomic status. Specifically, the health inequality is larger when the social groups present the following characteristics: male, people aged 31–40 years old, married, with non-local rural hukou, renting a house, farmers, three generations of people living together.

### 3.4. Robustness Test

In the analysis above, we report the basic result under the scenario that nearly all people keep wearing masks indoors according to the Government’s requirement and only one third of them keep wearing mask outdoors. However, there may be other scenarios which we could not ignore. It is also possible that not all people strictly comply with the rules and maybe less people wear mask outdoors. Therefore, we assume that when the compliance rate of wearing mask indoors is around one third and combing the situation of activities outdoors and circumstance differences, we calculate another risk weights of indoor activities and outdoor activities as 1 and 0.76, respectively. The risk of indoor activities is higher than that of outdoor activities in this condition. The result of this scenario is reported in the Appendix A. Comparing the results of two different scenarios with different weights settings, we find that the results of one-way ANOVA and regression models are consistent. The education level, income and vocation are still the statistically significant factors which have influence on the COVID19 exposure risk. The CV of the scenario in the robustness test is 1.49, which also shows great inequity. It means the basic discovery and conclusion are robust in this study.

## 4. Discussion

It is self-evident that the risks of exposure to COVID-19 are different for different activities and days. However, whether people with different social characteristics are at different risks of exposure to COVID-19 and the health inequities behind these differences are worth discussing.

Studies have shown that vulnerable groups, ethnic minorities and other groups in society have higher infection rates, less medical assistance and higher mortality rates after contracting COVID-19 [26,27,28,29,30,31,32]. Studies have also shown that these vulnerable and minority groups are hardest hit by COVID-19 [33,34]. This inequality is not a matter of chance. This structural inequality has caused widespread concern, and more and more people are calling for attention to these groups. That is, existing studies have focused strongly on pre- and post-infection inequalities. Now, our research shows that going back a step further, there are also some groups that are relatively at higher risk in terms of exposure. However, the groups at higher risk of COVID-19 exposure are different from these studies: they are not traditionally vulnerable groups.

On the whole, different groups of people demonstrate different activity patterns, leading to different exposure levels. However, when it comes to a specific influencing mechanism, the logic behind it needs further explanation.

People with higher education had higher levels of exposure risk than those with less than a junior high school education. Research showed that education level is negatively correlated with fear of being infected, which is consistent with our results to some extent [45,46]. The higher the level of education, the lower the fear of infection and the less likely they are to be afraid to go out, leading to a relatively high level of exposure. Coincidentally, some research demonstrates that education level is also positively correlated with the risk of COVID-19 infection [47]. That is, highly educated people are at a higher risk for both exposure and infection. However, a study pointed out that the higher the education level, the lower the actual infection rate [48]. A study also pointed out that the lower the education level, the higher the mortality rate of people infected with COVID-19 [49]. In addition, regarding the “side effects” of COVID-19, people with higher education levels also suffered fewer shocks (such as unemployment) [50], than people with lower education levels. These studies show that while people with more education are at higher risk of exposure and infection, this risk does not translate into reality impact. It is those with less education who suffer more infections, deaths and unemployment.

In terms of occupation, students are more exposed than civil servants. On the one hand, students are always aggregated in the classrooms with close contacts. On the other hand, it can be seen from our research that students’ frequent outdoor activities are an important reason for their high exposure risk. The reason for the large number of student activities is that students are more flexible in time arrangement and have higher social needs with their peers due to their attributes as young people. Other research points out that an important reason for the high risk of COVID-19 exposure among young people is that they think it will be fine to contact COVID-19 [51]. A cavalier attitude to the dangers of COVID-19 puts them at higher risk of exposure.

In terms of income, compared with the group with the monthly income ≤2000 yuan, the exposure risk of the group with the monthly income ≥30,001 yuan was higher (*p* < 0.01). The possible reason lies in the diversity of activities: the number of activities of people with a monthly income of more than 30,001 yuan is much higher than that of people with a monthly income of less than 2000 yuan.

Interestingly, Boyeong et al.’s measurements of COVID-19 exposure in US communities are the exact opposite of our results: Affluent neighborhoods in the United States have the lowest exposure, because in addition to being able to reduce contact by moving to a second home in the suburbs, residents in these neighborhoods have the option of working remotely. Poor minority communities are at greater risk of exposure because they tend to work in basic service jobs where the need to survive prevents them from strictly enforcing government’s stay-at-home orders [52]. The reason for the completely opposite results of the empirical studies in the two countries may be that the socioeconomic context and the definition of high-income groups are different. Boyeong et al.’s basic analyzing unit is the community, whereas ours is the individual. The American high income groups belongs to wealthy neighborhoods and live far away from the poor ones. The situation is totally different in the megacity in China. In addition, residents’ living habit and activity features of different social groups in China are also different from that of the American residents. We found a robust result even when we applied different weights during the calculations. It may be because most of the respondents’ activities are indoors in the megacity, and the proportion of indoor activities overweight the outdoor activities. Besides, this paper aims to explore the relative differences of exposure risks among respondents, and both CVs of different scenarios are larger than 1, which means great diversity of different social groups. The comparative result is not hampered based on different weights analysis.

Compared to existing literature, this study makes contributions in the following aspects: first, most studies aggregated on the research of infection rate and death rate of COVID-19 and other types of epidemics. This research explores the risk of exposure to COVID-19 under the condition of uncertainty and analyzes the impact of different individuals’ activities and social characteristics on their exposure to COVID-19. Second, existing studies usually focus on particular groups of people such as medical staffs and the infected people, and the general public under the risk of becoming infected are paid less attention. This study focuses on the general public and reveals the COVID-19 exposure risk of general public during their different activities at different time. Third, studies on the inequality of COVID-19 or other epidemics are less, this study measures the inequality of the risk of exposure to COVID-19 in and between different social groups, which could enlighten the governments to take effective measures to reduce the infection rate from the perspective of exposure risk.

However, there are still some limitations in this paper. First, although the professional structure of Beijing residents was considered in the design and collection of questionnaires, the questionnaires number was still small in general. Second, based on Beijing’s compulsive “wear a mask” policy, we calculate the exposure risks on the assumption that nearly everyone took strict self-protection measures and wore masks correctly indoors and one third of people also wore masks outside. Different compliance rates of wearing masks indoors and outdoors could lead to different sets of weights which would lead to different values of exposure risks. Although the conclusions of influencing factors of COVID-19 and inequity under uncertainty are robust, there are inevitable value deviations. It needs further and deeper exploration under different circumstances. Lastly, the factors affecting individuals’ exposure risk of COVID-19 in this paper are not fully considered. People with poor immunity and basic diseases are susceptible and have higher chance of becoming infected, when compared with other people, even if they are exposed in the same situation. In the external environment, the spread of the new coronavirus will be affected by meteorological factors such as temperature and humidity [53]. However, in this study, we did not consider individual health condition and the impact of external environmental factors on the exposure levels. The deficiencies of this study also provide a direction for further research.

## 5. Conclusions

### 5.1. Differences in Exposure Risk among Activities

Affected by time and frequency, the exposure risk of each activity is different. First, the exposure risk was always highest for work activities, regardless of the frequency of activity. Second, regardless of the frequency of activity, low-exposure activities, such as dining out, commuting, and picking up kids, did not differ much between weekdays and rest days. Leisure, personal affairs, and socializing are activities with high exposure risk, and the exposure risk is higher on work days than on rest days. Finally, after considering the frequency of activity, dining out, commuting, and picking up kids ranked significantly higher on work days, while leisure, personal affairs and socializing ranked lower.

In terms of the total exposure risk within one week, the exposure risk of subsistence activities was the highest, the exposure risk of living activities was in the middle, and the exposure risk of leisure activities was relatively the lowest. Specifically, the exposure risks of these eight activities in one week, in descending order, are: work, commuting, dining out, picking up children, leisure, personal affairs, life and shopping, and social contact.

### 5.2. Differences in Exposure Risk among Social Groups

Education level, occupation and income passed the statistical test and showed significant differences of COVID-19 exposure risks. In terms of the education level, residents with master’s degree or above have the highest exposure risk, followed by those with bachelor’s degree. People with a junior high school education or less had the lowest exposure risk. In terms of occupational types, the exposure risk of students is the highest. As for the income level, residents with a monthly income of more than 30,001 yuan had the greatest exposure risk, followed by those with a monthly income of 10,001–15,000 yuan. 

### 5.3. Inequality of Exposure Risk

The coefficients of variation of exposure risk of the whole sample under two scenarios are 1.46 and 1.49, indicating that diversities in exposure risk among residents and health inequity existed. The health inequities are higher on weekdays than on rest days. In terms of activity type, work presents the highest degree of health inequality. This is because each job varies greatly in its working environment, requirements, working hours, population density and mobility. In addition, other activities also present great differences of the exposure risks, expect for the dining out activity.

Health inequalities also exist within groups of different socioeconomic status. The social groups, characterized as male, 31~40 ages, with a high school education degree, married, non-local agricultural hukou, renting houses, farmers, working in private enterprises, income more than 30,001 yuan, are demonstrating higher inequity within the group with larger coefficients of variation of exposure risk. As for people with the same attribute, we have sketched out a portrait of a person who is suffering from the highest level of health inequality within the group: male, 31–40 years old, married, with a rural hukou, rented housing, farmers, three generations of cohabitation.

## Figures and Tables

**Table 1:** Demographic characteristics of the sample.

Variable	n	Percentage (%)	Variable	n	Percentage (%)
**Age**			**Occupation**		
≤18	12	1.59	Civil servant	26	3.45
19–30	430	57.03	Public institutions	71	9.42
31–40	200	26.53	State-owned enterprise	128	16.98
41–50	81	10.74	Private-owned enterprise	260	34.48
51–60	21	2.79	Foreign-owned enterprise	39	5.17
≥61	10	1.33	Individual business	8	1.06
**Gender**			Freelancer	42	5.57
Male	398	52.79	Farmer	16	2.12
Female	356	47.21	Student	75	9.95
**Education**			Unemployed	12	1.59
≤Junior high school	68	9.02	Retirees	18	2.39
High school	110	14.59	Others	59	7.82
Junior college	119	15.78	**Income (yuan)**		
Undergraduate	303	40.19	≤2000	71	9.42
Postgraduate	154	20.42	2001–3500	102	13.53
**Marital status**			3501–5000	111	14.72
Married	374	49.6	5001–10,000	223	29.58
Divorced/widowed	18	2.39	10,001–15,000	146	19.36
Unmarried	362	48.01	15,001–20,000	54	7.16
**Hukou**			20,001–25,000	16	2.12
Beijing urban	232	30.77	25,001–30,000	14	1.86
Beijing agricultural	54	7.16	≥30,001	17	2.25
Non-local urban	182	24.14	**Family structure**		
Non-local agricultural	286	37.93	Live alone	335	44.43
**House Ownership**			With parents	96	12.73
Self-purchased	195	25.86	With partner	133	17.64
Rented	395	52.39	With partner and children	128	16.98
Staff apartment	85	11.27	With three generations or more	62	8.22
Others	79	10.48	—	—	—

**Table 2:** Exposure risk level of each activity.

	Without Considering the Frequency of Activity		Considering the Frequency of Activity	
Activity	Work Days	Rest Days	Work Days	Rest Days
Commuting	1396	597	5137	454
Dining out	1406	856	4323	1731
Work	4025	2996	15,349	2098
Personal affairs	2198	1266	3179	1844
Shopping	1493	1106	2697	1624
Picking up kids	715	328	3577	328
Leisure	2155	1358	3351	2002
Socializing	1665	1302	2677	1849

**Table 3:** Exposure risk level of all activities within a week.

Activity	n	Min	Max	Median	Average	Standard Deviation
Commuting	529	30	20,608	3827	4964	4751
Dining out	326	60	28,800	2861	4120	3782
Work	404	60	390,000	6480	14,552	28,183
Personal affairs	254	100	20,720	1867	2979	3057
Shopping	380	70	29,400	1460	2868	3854
Picking up kids	92	50	22,920	2150	3334	4199
Leisure	228	160	35,000	1920	3145	3974
Socializing	188	70	24,320	1705	2755	3576
Total exposure	754	147	446,025	11,556	17,555	25,719

**Table 4:** Exposure level of three kinds of activities.

Activity	Work Days				Rest Days				A Total of a Week			
Type	Min	Max	Median	Mean	Min	Max	Median	Mean	Min	Max	Median	Mean
(1)	147	392,208	8538	12,901	30	17,280	608	1454	30	392,208	8277	13,165
(2)	60	39,017	3987	5564	50	19,272	1752	2623	50	52,272	4047	6255
(3)	140	42,000	2280	3923	70	17,800	1520	2624	70	58,480	2680	4201

Note: (1), (2) and (3) respectively represent survival activities, daily activities and leisure activities.

**Table 5:** Exposure risk level of different social groups.

Variable	Min	Max	Median	Average	Variable	Min	Max	Median	Average
**Gender**					**Occupation**				
Male	147	446,025	11,200	17,938	Civil servant	325	87,792	5156	12,985
Female	147	189,188	11,591	17,125	Public institutions	178	91,191	12,268	15,943
**Age**					State-owned enterprise	178	189,188	10,483	17,851
≤18	325	35,385	9361	13,758	Private-owned enterprise	147	446,025	14,351	19,308
19–30	147	116,080	13,275	17,673	Foreign-owned enterprise	470	53,474	10,516	12,952
31–40	147	446,025	11,148	20,465	Individual business	178	19,155	2967	6698
41–50	147	73,744	8413	13,276	Freelancer	147	46,119	6724	11,152
51–60	340	86,491	2640	10,948	Farmer	442	52,891	3322	9281
≥61	840	22,680	5290	7364	Student	420	116,080	18,240	27,071
**Education**					Unemployed	790	17,694	5911	7603
≤Junior high school	147	33,600	2100	5546	Retirees	320	31,080	3020	7708
High school	147	446,025	5925	17,175	Others	147	172,859	9458	17,388
Junior college	178	86,491	9267	15,581	**Income (yuan)**				
Undergraduate	147	189,188	14,476	19,004	≤2000	324	87,360	7180	15,481
Postgraduate	294	116,080	15,210	21,803	2001–3500	147	116,080	9361	16,747
**Marital status**					3501–5000	147	86,491	7390	11,981
Married	147	446,025	9054	15,716	5001–10,000	177	195,152	13,199	18,253
Divorced/widowed	442	86,491	7935	16,739	10,001–15,000	294	172,859	14,168	20,348
Unmarried	238	189,188	14,650	19,495	15,001–20,000	460	64,966	13,149	15,384
**Hukou**					20,001–25,000	3230	50,412	14,840	19,338
Beijing urban	294	172,859	12,237	17,532	25,001–30,000	3240	31,960	9725	11,449
Beijing agricultural	147	73,744	4922	11,400	≥30,001	1827	446,025	19,500	44,563
Non-local urban	294	195,152	12,577	19,822	**Family structure**				
Non-local agricultural	147	446,025	11,159	17,293	Live alone	147	189,188	12,499	18,101
**House Ownership**					With parents	324	195,152	11,421	18,506
Self-purchased	147	73,744	10,300	14,308	With partner	441	87,792	12,659	16,261
Rented	147	195,152	13,316	18,392	With partner and children	147	64,622	9586	13,855
Staff apartment	294	129,600	11,150	17,962	With three generations or more	147	446,025	9918	23,543
Others	147	446,025	8050	20,947	—	—	—	—	—

**Table 6:** Result of one-way ANOVA analysis.

Variable	F	Prob > F	[95% Conf. Interval]	
Gender	0.02	0.89	0	0.01
Age	2.52	0.02	0	0.03
Education	18.39	0	0	0.08
Marriage	9.54	0	0	0.02
Occupation	4.57	0.01	0	0.06
Income	2.78	0	0	0.08
Hukou	2.89	0.06	0	0.02
House ownership	1.46	0.23	0	0.02
Family structure	0.79	0.53	0	0.02

**Table 7:** Regression results of influencing factor analysis.

Variables	Coefficient	S.D.	T	95% Confidence Interval	
**Education (Reference group: Junior high school and below)**					
High school	5360 ***	2041	2.63	1353	9367
Junior college	9225 ***	2011	4.69	5276	13,173
Undergraduate	11,729 ***	1775	6.61	8243	15,214
Postgraduate	14,226 ***	1926	7.38	10,444	18,008
**Occupation (Reference group: Civil servants)**					
Public institution	3791	3090	1.23	−2275	9859
State-owned enterprise	3905	2900	1.35	−1788	9599
Private-owned enterprise	5183	2773	1.87	−260	10,627
Foreign-owned enterprise	1288	3413	0.38	−5412	7898
Individual business	−4844	5451	−0.89	−15,546	5856
Freelancer	−413	3364	−0.12	−7018	6191
Farmer	−2454	4284	−0.57	−10,864	5955
Student	11,164 **	3068	3.64	5140	17,188
Unemployed	−3983	4705	−0.85	−13,221	5253
Retirees	−3856	4134	−0.93	−11,972	4259
Others	1948	3173	0.61	−4281	8179
**Income (Reference group: ≤2000 Yuan)**					
2001–3500	−32	2117	−0.02	−4189	4124
3501–5000	−2614	2082	−1.26	−6702	1472
5001–10,000	1800	1866	0.96	−1865	5465
10,001–15,000	4026	1982	1.03	134	7917
15,001–20,000	874	2473	0.35	−3982	5730
20,001–25,000	5096	3791	1.34	−2346	12,540
25,001–30,000	−2792	4006	−0.7	−10,657	5073
≥30,001	7050 **	3699	1.91	−211	14,313
**Control variables**					
Gender	456	1967	0.44	−3347	4375
Age	875	1606	1.44	−1227	5077
Marital status	1057	1306	1.05	−1188	3941
Hukou	−475	896	−0.37	−2090	1429
House Ownership	725	1868	1.26	−1319	6015
Family structure	1302	805	1.11	−362	1297
Constant	−825	8618	−0.94	−24,978	8860

Note: *** *p* < 0.01, ** *p* < 0.05, * *p* < 0.1.

**Table 8:** Coefficient of variation of residents in all social groups.

Variable	CV	Variable	CV
**Gender**		**Occupation**	
Male	l.67	Civil servants	1.55
Female	1.17	Public institutions	0.99
**Age**		State-owned enterprises	1.37
≤18	1.05	Private enterprises	1.65
19–30	l.02	Foreign enterprises	0.96
31–40	1.98	Individual business	1.10
41–50	1.14	Freelancer	0.97
51–60	1.80	Farmers	l.69
≥61	0.94	Students	0.98
**Education**		Unemployed	0.72
≤Junior high school	1.40	Retirees	1.18
High school	2.82	Others	l.63
Junior college	1.00	**Income(yuan)**	
Undergraduate	1.09	≤2000	1.24
Postgraduate	0.96	2001–3500	1.38
**Marital status**		3501–5000	1.14
Married	1.91	5001–10,000	1.29
Divorced/widowed	1.30	10,001–15,000	1.07
Unmarried	l.06	15,001–20,000	0.88
**Hukou**		20,001–25,000	0.65
Beijing urban	l.18	25,001–30,000	0.70
Beijing agricultural	1.40	≥30,001	2.34
Non-local urban	1.32	**Family structure**	
Non-local agricultural	1.75	Live alone	1.23
**House Ownership**		With parents	1.32
Self-purchased House	0.99	With partner	0.94
Rented house	1.59	With partner and children	l.01
Staff apartment	1.13	With three generations or more	2.53

**Figure A1:**
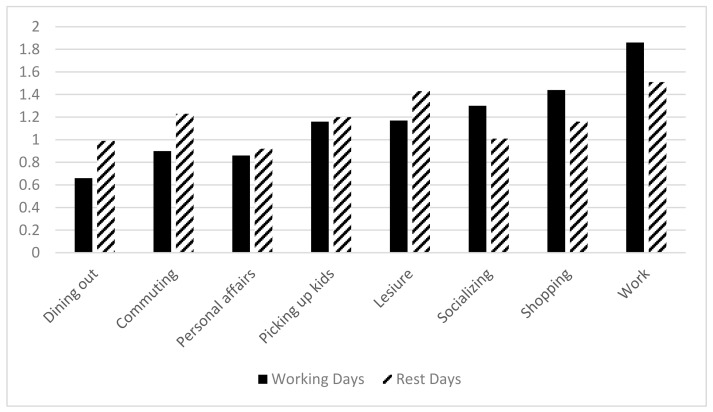
Coefficient of variation of different activities.

## Data Availability

All data included in this study are available upon request by contact with the corresponding author.

## Appendix A

**Table A1:** Exposure level of each activity.

	Without Considering the Frequency of Activity		Considering the Frequency of Activity	
Activity	Work Days	Rest Days	Work Days	Rest Days
Commuting	530	227	1952	172
Dining out	1406	757	4323	1518
Work	4025	2997	15,349	2098
Personal affairs	1146	830	1649	1213
Shopping	914	811	1703	1197
Picking up kids	715	190	3577	190
Leisure	1268	965	2021	1434
Socializing	1058	939	1731	1322

**Table A2:** Exposure level of all activities within a week.

Activity	n	Min	Max	Median	Mean	Standard Deviation
Commuting	529	12	7831	1454	1886	1805
Dining out	326	60	21,360	2683	3977	3625
Work	404	60	390,000	6480	14,552	28,183
Personal affairs	254	75	11,157	1171	1735	1832
Shopping	380	45	24,787	950	1930	2732
Picking up kids	92	25	9354	1355	2090	2099
Leisure	228	135	22,600	1121	2060	2719
Socializing	188	45	16,384	1092	1850	2394
Total exposure	754	56	436,561	7475	13,736	24,223

**Table A3:** Exposure level of three kinds of activities.

Activity	Work Days				Rest Days				A Total of a Week			
Type	Min	Max	Median	Mean	Min	Max	Median	Mean	Min	Max	Median	Mean
(1)	30	304,208	9120	11,132	15	10,080	456	1003	120	390,839	4755	10,645
(2)	60	36,707	3987	4534	15	18,743	1542	2311	250	36,707	2895	4763
(3)	140	42,000	2280	2913	61	12,845	1254	2421	450	37,772	1603	2780

Note: (1), (2) and (3) respectively represent survival activities, daily activities and leisure activities.

**Table A4:** Exposure level of different social groups.

Variable	Min	Max	Median	Mean	Variable	Min	Max	Median	Mean
**Gender**					**Occupation**				
Male	56	436,521	6933	13,919	Civil servant	123	57,085	4011	8593
Female	56	146,973	8066	13,532	Public institutions	67	88,511	8612	12,778
**Age**					State-owned enterprise	67	142,973	6898	13,512
≤18	123	33,600	5240	10,981	Private-owned enterprise	56	436,561	8928	14,501
19–30	56	111,814	8258	13,658	Foreign-owned enterprise	280	45,461	6082	8734
31–40	56	436,561	7081	10,269	Individual business	67	12,242	2101	3974
41–50	56	73,645	4690	10,397	Freelancer	56	38,268	4943	8193
51–60	168	83,635	2002	9344	Farmer	168	52,835	1842	7952
≥61	542	20,944	4992	6008	Student	269	111,814	16,374	24,603
**Education**					Unemployed	641	16,667	4045	5762
≤Junior high school	56	33,600	1300	3891	Retirees	146	30,894	2524	6692
High school	56	436,561	3821	15,016	Others	56	172,859	4282	13,748
Junior college	67	86,435	6407	11,708	**Income (yuan)**				
Undergraduate	56	172,859	9257	14,093	≤2000	123	87,360	5243	13,147
Postgraduate	112	111,814	11,133	18,034	2001–3500	56	111,814	7237	14,137
**Marital status**					3501–5000	56	86,435	4767	9348
Married	56	436,560	5595	12,261	5001–10,000	67	195,058	7445	13,723
Divorced/widowed	168	86,435	6897	13,986	10,001–15,000	112	172,859	9944	15,451
Unmarried	91	142,973	9773	15,247	15,001–20,000	361	56,302	7894	11,506
**Hukou**					20,001–25,000	1431	49,028	10,939	14,843
Beijing urban	112	172,859	8275	6983	25,001–30,000	1711	24,498	7074	8125
Beijing agricultural	56	73,645	2881	9133	≥30,001	2760	37,992	12,505	16,503
Non-local urban	112	195,058	8086	15,222	**Family structure**				
Non-local agricultural	56	436,560	14,051	13,403	Live alone	56	172,859	8029	14,267
**House Ownership**					With parents	123	195,058	7390	147,456
Self-purchased	56	73,645	6566	10,737	With partner	168	87,360	7286	11,249
Rented	56	195,058	8259	14,148	With partner and children	56	61,909	6392	10,758
Staff apartment	112	129,600	7918	14,629	With three generations or more	56	436,561	6359	20,785
Others	56	436,560	4613	18,115	—	—	—	—	—

**Table A5:** Result of one-way ANOVA analysis.

Variable	F	Prob > F	[95% Conf. Interval]	
Gender	0.05	0.82	0	0.01
Age	1.12	0.35	0	0.03
Education	4.4	0	0	0.08
Marriage	1.4	0.25	0	0.02
Occupation	2.13	0.01	0	0.06
Income	5.8	0	0	0.00
Hukou	0.91	0.44	0	0.01
House ownership	1.94	0.12	0	0.03
Family structure	2.24	0.06	0	0.03

**Table A6:** Regression results of influencing factor analysis.

Variables	Coefficient	S.D.	T	95% Confidence Interval	
**Education (Control group: Junior high school and below)**					
High school	11,255.91	3786.166	2.97	3822.685	18,689.14
Junior college	6221.725	4025.842	1.65	−1682.048	14,125.5
Undergraduate	7447.938	3848.758	1.94	−108.1725	15,004.05
Postgraduate	5192.66	4320.455	1.20	−3289.516	13,674.84
**Occupation (Control group: Civil servants)**					
Public institution	206.1443	5547.156	0.04	−10,684.36	11,096.65
State-owned enterprise	−99.67065	5231.173	−0.02	−10,369.82	10,170.48
Private-owned enterprise	1351.659	5174	0.26	−8806.247	11,509.56
Foreign-owned enterprise	−6749.391	6172.625	−1.09	−18,867.86	5369.075
Individual business	−6347.349	9573.831	−0.66	−25,365.26	5418.57
Freelancer	−3109.181	6088.612	−0.51	−15,062.71	8844.346
Farmer	−4272.89	8176.362	−0.52	−20,325.21	11,779.43
Student	17,361	6007.563	2.89	5566.592	29,155.4
Unemployed	−8696.053	8551.988	−1.02	−25,485.83	8093.721
Retirees	−7558.532	8494.988	−0.89	−24,236.4	9119.335
Others	2572.403	5807.623	0.44	−8829.469	13,974.27
**Income (Control group: ≤2000 Yuan)**					
2001–3500	−1036.404	3732.591	−0.28	−8364.45	6291.642
3501–5000	−2077.903	4002.277	−0.52	−9935.412	5779.606
5001–10,000	3877.221	3947.552	0.98	−3872.849	11,627.29
10,001–15,000	5582.416	4251.809	1.31	−2764.99	13,929.82
15,001–20,000	1825.864	5045.633	0.36	−8080.023	11,731.75
20,001–25,000	5041.571	7045.96	0.72	−8791.479	18,874.62
25,001–30,000	−1184.71	7435.496	−0.16	−15,782.52	13,413.1
≥30,001	−1184.71	7435.496	−0.16	−15,782.52	13,413.1
**Control variables**					
Gender	718.2819	1821.083	0.39	−2856.977	4293.541
Age	1658.5	1499.84	1.11	−1286.076	4603.076
Marital status	902.3923	1198.797	0.75	−1451.157	3255.942
Hukou	−441.1183	802.6724	−0.55	−2016.973	1134.736
House Ownership	2459.07	1048.734	2.34	400.1329	4518.008
Family structure	1126.592	742.0304	1.52	−330.206	2583.391
**Constant**	−9973.052	7839.551	−1.27	−25364.13	5418.022

**Table A7:** Coefficient of variation of residents in all groups.

Variable	CV	Variable	CV
**Gender**		**Occupation**	
Male	2.06	Civil servants	1.58
Female	1.32	Public institutions	1.13
**Age**		State-owned enterprises	1.53
≤18	1.07	Private enterprises	2.14
19–30	1.33	Foreign enterprises	1.09
31–40	2.11	Individual business	1.10
41–50	1.03	Freelancer	1.03
51–60	1.77	Farmers	2.0
≥61	0.88	Students	0.99
**Education**		Unemployed	1.85
≤Junior high school	1.33	Retirees	1.26
High school	2.91	Others	2.09
Junior college	1.11	**Income(yuan)**	
Undergraduate	1.21	≤2000	1.51
Postgraduate	0.99	2001–3500	1.33
**Marital status**		3501–5000	1.55
Married	1.77	5001–10,000	1.33
Divorced/widowed	1.43	10,001–15000	0.98
Unmarried	1.22	15,001–20,000	0.88
**Hukou**		20,001–25,000	0.81
Beijing urban	l.40	25,001–30,000	0.74
Beijing agricultural	1.65	≥30,001	2.21
Non-local urban	1.52	**Family structure**	
Non-local agricultural	2.17	Live alone	1.4
**House Ownership**		With parents	1.62
Self-purchased House	1.16	With partner	1.14
Rented house	1.38	With partner and children	2.82
Staff apartment	1.32	With three generations or more	1.76
